# Reported Levels of Upset in Youth After Routine Trauma Screening at Mental Health Clinics

**DOI:** 10.1001/jamanetworkopen.2019.4003

**Published:** 2019-05-17

**Authors:** Ane-Marthe Solheim Skar, Silje Mørup Ormhaug, Tine K. Jensen

**Affiliations:** 1 Norwegian Centre for Violence and Traumatic Stress Studies, Oslo, Norway; 2 Department of Psychology, University of Oslo, Oslo, Norway

## Abstract

**Importance:**

Clinical guidelines recommend routine screening for trauma at youth mental health clinics. However, many clinicians are concerned that screening may be upsetting both for youths who have been exposed to trauma and for those who have not.

**Objectives:**

To investigate levels of upset following routine trauma screening and whether type of trauma and symptoms of posttraumatic stress were associated with level of upset.

**Design, Setting, and Participants:**

Survey study in which data from routine screening for trauma exposure and trauma-related symptoms were collected from January 1, 2015, to December 31, 2017. Nearly half (n = 40) of all youth mental health clinics in Norway submitted survey data. Participants included youths aged 6 to 18 years referred to treatment.

**Main Outcomes and Measures:**

The trauma screening inventory, Child & Adolescent Trauma Screening Questionnaire, was administered. Self-reported levels of upset after initial screening were scored on a scale ranging from 1 (not upsetting) to 7 (very upsetting).

**Results:**

Among 10 157 youths aged 6 to 18 years (mean [SD] age, 13.0 [3.1] years; 5320 [55.0%] female), surveyed, 8021 (79.1%) reported exposure to at least 1 potentially traumatizing event and a mean (SD) of 2.44 (2.27) different types of events (range, 0-15). Only 453 participants (4.5%) reported high levels of upset, while 2757 participants (27.2%) reported moderate levels of upset and 6942 participants (68.4%) reported no or low levels of upset. Youths who had been exposed to any type of traumatic event reported significantly higher levels of upset compared with those reporting no trauma exposure, and a higher number of reported traumatic events was associated with a higher level of upset (point estimate, 0.23; 95% CI, 0.22-0.24; *P* < .001). Among participants who reported trauma, exposure to sexual abuse (point estimate, 0.87; 95% CI, 0.77-0.95; *P* < .001) and higher levels of posttraumatic stress symptoms (point estimate, 0.05; 95% CI, 0.04-0.05; *P* < .001) were associated with higher levels of upset.

**Conclusions and Relevance:**

A high level of trauma exposure was found among youths referred to youth mental health clinics, but trauma screening was well tolerated, with most participants reporting no or low levels of upset. These findings can be used to support therapists and health care leaders in implementing routine trauma screening to better identify youths who are in need of trauma-focused treatment.

## Key Points

**Question:**

Do youths find routine trauma screening in youth mental health clinics upsetting?

**Findings:**

In this survey study of 10 157 patients aged 6 to 18 years in Norway who completed routine trauma screening at youth mental health clinics, high rates of trauma exposure were reported. Most participants (68.4%) reported no or low levels of upset after screening, 27.2% reported moderate levels, and 4.5% reported high levels.

**Meaning:**

Routine trauma screening is recommended in youth mental health services, as the rate of exposure is high; although most patients do not find routine trauma screening very upsetting, some youths experience some kind of distress, and it is important that the therapist is attentive and supporting.

## Teaser

This survey study investigates levels of upset reported by youths after routine trauma screening in mental health clinics in Norway.

## Introduction

Childhood exposure to trauma is high. Two 2014 population-based studies suggested that most children throughout the world have experienced threat-related events, including child abuse, sexual abuse, family and community violence, natural disasters, serious unintentional injuries, sudden or violent loss of loved ones, and war.^1,2^ Rates are even higher among clinical populations that include children in psychiatric inpatient and child welfare settings.^3,4^ Although not all youths who are exposed to trauma develop mental health problems, exposure to trauma and other stressors is associated with the onset of many childhood disorders in general and posttraumatic stress disorder (PTSD) in particular.^5,6,7^ Despite this, when youths are referred to treatment, trauma exposure is often not mentioned. For instance, the Norwegian Patient Registry, a national registry that includes information about all patients who receive specialist health services in Norway, revealed that only 1.8% of referrals to youth mental health clinics were associated with stress reactions after a traumatic event.^8^ These numbers are in sharp contrast with a 2006 study^9^ of a representative clinical sample in which 60.2% of children reported they had been exposed to abuse or neglect. There may be several reasons why youths who have been exposed to trauma are not identified in youth mental health clinics. For instance, children with sexual abuse experiences often do not disclose their abuse,^10^ and therapists may not link children’s problems to trauma if the therapists do not ask about abuse. This is alarming, since an increase in time from incident to disclosure among adult female survivors of childhood sexual abuse has been found to be significantly associated with an increase in symptoms of posttraumatic stress, anxiety, and depression,^11^ underscoring the importance of early detection. In addition, PTSD is an internalizing disorder, and adults who interact with these children, such as caregivers, teachers, and mental health workers, may not detect children’s experiences. For instance, a 2011 study^12^ reported that children with externalizing disorders were more likely to receive services compared with children with internalizing disorders. Moreover, trauma-focused interventions are recommended for youth who experience posttraumatic stress after trauma exposure.^13^ Therefore, it is important that services have routine procedures to help identify youth who have been exposed to traumatizing events and recommend the appropriate interventions.

Although routine screening for posttraumatic stress symptoms is recommended in the National Institute for Health and Care Excellence guidance,^13^ health authorities as well as leaders within mental health care systems and mental health workers occasionally express concern about the potential harm of asking youths about exposure to trauma.^14,15^ Since PTSD is characterized by easily activated stress responses when thinking or talking about the trauma,^16,17^ some degree of emotional activation could be expected when people with PTSD are reminded of the trauma through a questionnaire. In addition, it is likely that youths who have been exposed to interpersonal trauma could become upset because of trauma-related shame.^18,19^ Therefore, a crucial question is whether asking about trauma exposure is upsetting for youths and whether it may be particularly upsetting for youths with higher symptom levels and more interpersonal trauma experiences. Research on population-based samples suggests that less than 6% of children report upset following questions about trauma exposure,^20,21^ but studies from clinical samples are lacking, to our knowledge.

We postulate that rates of trauma exposure among patients at youth mental health clinics are high and that most youths will not find trauma screening upsetting. Additionally, we hypothesize that youths who have been exposed to interpersonal traumas, such as sexual abuse and domestic violence, will report higher levels of upset compared with those who have been exposed to noninterpersonal traumas and that youths with higher levels of posttraumatic stress will report higher levels of upset.

## Methods

### Procedure

The data in this study were derived from an ongoing project in which trauma-focused cognitive behavioral therapy is being implemented in youth mental health services in Norway. As part of the implementation, youths are routinely screened for trauma exposure and posttraumatic stress symptoms at intake. The inclusion criterion was being aged 6 to 18 years. The implementation project started in 2012, and by 2015, of the approximately 85 youth mental health clinics in Norway, 40 had implemented trauma-focused cognitive behavioral therapy. This study included data collected from January 1, 2015, to December 31, 2017. The study was approved by the Norwegian Regional Committee for Medical and Health Research Ethics, and a waiver of consent was granted because the data obtained were deidentified.

### Outcome Measures

#### Trauma Experiences

The participants completed a screening inventory^22^ including 15 traumatic events, administered as self-reported or as an interview. The instructions read [in Norwegian], “In order to be able to help you in the best possible way, it is helpful for us to know a little bit about what you have experienced. In the list below, you will find questions about different events that youths can experience. If this happened to you, and you felt scared, confused, or helpless, mark ‘yes.’ If there is a question you don’t want to answer, mark ‘pass.’” The inventory included questions such as, “Have you ever been in a serious accident (eg, car accident)?”; “Have you ever seen anyone in your family being hit or fight or attack each other?”; and “Has anyone touched your private parts or have you been forced to touch others’ private parts (eg, penis or vagina, buttocks, or breasts)?” Participants responded yes, no, or pass. Marking pass was associated with higher upset scores and was therefore coded as yes. Items within the same trauma category were collapsed to find the prevalence of exposure to domestic violence (ie, witnessing violence in the family or physical abuse from a caregiver), community violence (ie, war and terror, kidnapping, witnessing or direct exposure to violence outside the family, or exposure to severe bullying or threats), sexual abuse (ie, forced to take pictures of private parts, someone forcefully touching private parts or forced to touch others’ private parts, or forced intercourse), and noninterpersonal traumas (ie, serious accidents, natural disaster, medical trauma, or sudden death of a close person). In addition, a final category of other scary event was included in the descriptions but not in the linear mixed models, as we did not know what experiences this category included. The number of traumas experienced by the participant was summed to get a total score.

#### Symptoms of Posttraumatic Stress

Symptoms of PTSD were assessed with the Child and Adolescent Trauma Screening Questionnaire,^23^ which assess the frequency of all posttraumatic stress symptoms within the previous 2 weeks for youths who have experienced at least 1 potentially traumatizing experience. It is based on the diagnostic criteria in *Diagnostic and Statistical Manual of Mental Disorders* (Fifth Edition) and consists of 20 symptom items and 5 functional impairment items. A total symptom severity scale score from 0 to 60 was calculated. The screening cutoff was 15. Cronbach α for the total scale was 0.93.

#### Upset Following Trauma Screening

To assess for level of upset after the trauma screening, the first item from the Reaction Questionnaire^24^ was included: “Did you find it upsetting or stressful to answer these questions?” Participants responded on a visual analog scale ranging from 1 (not at all) to 7 (very). Upsetting or stressful will hereafter be referred to as *upset*. A score of 1 to 2 indicates no or minimal levels of upset, 3 to 5 indicates moderate levels of upset, and 6 to 7 indicates high levels of upset.^24^

### Statistical Analysis

Sample characteristics were analyzed with descriptive statistics, and between-group comparisons were conducted with independent-sample *t* tests. Factors associated with reported levels of upset were analyzed with linear mixed models, with participants nested within clinic. Three models were tested. The first model included number of traumatic events, the second model included types of trauma exposure, and the third model included posttraumatic stress symptoms as the associated variable. Because sex and age have been found to be associated with levels of symptoms of PTSD,^5^ these 2 variables were included in all 3 models as control variables. To test significance of differences in estimates for trauma exposure, linear hypotheses were run and were adjusted with Holm correction. All tests were 2-sided, and the level of significance was set to less than .05. Initial analyses were run in PASW Statistics version 22.0 (IBM SPSS Statistics). Linear mixed models and linear hypotheses were conducted using the nlme and car packages in R statistical software version 3.4.4 (The R Project for Statistical Computing). In all analyses that included data from the trauma screening inventory or the PTSD inventory, participants with a total of 5 or fewer missing values from each of the scales were included in analyses; therefore, the total number of participants varies in the different analyses.

## Results

### Study Population

The total sample included 10 157 youths. They were aged 6 to 18 years, with a mean (SD) age of 13.0 (3.1) years (1001 participants did not provide information about age). There were 5320 female participants (55.0%) (489 participants did not provide information about sex).

### Trauma Exposure

A total of 0.95% (range, 0.49%-2.88%) of the responses were marked pass, and were counted as yes. In the sample, 8021 participants (79.1%) reported exposure to at least 1 potentially traumatizing event. The mean (SD) number of traumas reported was 2.44 (2.27) (range, 0-15). Exposure to noninterpersonal traumas was the most commonly reported event, reported by 6007 participants (59.2%), and exposure to violence outside the family was reported by 5016 participants (49.4%). About one-quarter of the sample reported exposure to domestic violence (2715 [26.8%]) or other traumas (2451 [25.9%]). Exposure to sexual abuse was reported by 1457 youths (14.4%). Among the 8021 youths who had been exposed to trauma, the mean (SD) number of different traumatic events was 3.34 (2.29) (range, 1-15).

### Level of Upset

The mean (SD) level of self-reported upset was low (2.18 [1.56]). In the total sample, 6942 participants (68.4%) reported no or low levels of upset, 2757 participants (27.2%) reported moderate levels of upset, and 453 participants (4.5%) reported high levels of upset. When comparing levels of upset, we found that youths who had been exposed to trauma reported significantly higher mean (SD) levels of upset compared with youths who had not been exposed to trauma (2.34 [1.62] vs 1.56 [1.11]). In the group with trauma exposure, 63.5% (5103 of 8021) reported minimal levels of upset, and in the group without trauma exposure, 86.5% (1833 of 2119) reported minimal levels of upset. Table 1 presents details about levels of upset across the different trauma types.

**Table 1.** Level of Upset Among Youth Who Have Been Exposed to Trauma vs Youth Who Have Not Been Exposed to Potentially Traumatic Experiences

Exposure to Trauma Type	No. (%)				χ^2^	*P* Value
	All	Low Upset (Score, 1-2)	Moderate Upset (Score, 3-5)	High Upset (Score, 6-7)		
Trauma exposure (n = 10 140)						
No	2119 (20.9)	1833 (86.5)	254 (12.0)	32 (1.5)	407.14	<.001
Yes	8021 (79.1)	5103 (63.5)	2499 (31.2)	419 (5.2)		
Domestic violence (n = 10 117)						
No	7402 (73.2)	5467 (73.9)	1695 (22.9)	240 (3.2)	392.32	<.001
Yes	2715 (26.8)	1454 (53.6)	1052 (38.7)	209 (7.7)		
Community violence (n = 10 150)						
No	5134 (50.6)	4039 (78.7)	961 (18.7)	134 (2.6)	513.75	<.001
Yes	5016 (49.4)	2901 (57.8)	1796 (35.8)	319 (6.4)		
Sexual abuse (n = 10 118)						
No	8661 (85.6)	6322 (73.0)	2049 (23.7)	290 (3.3)	608.71	<.001
Yes	1457 (14.4)	603 (41.4)	695 (47.7)	159 (10.9)		
Noninterpersonal violence (n = 10 149)						
No	4142 (40.8)	3183 (76.8)	815 (19.7)	144 (3.5)	232.76	<.001
Yes	6007 (59.2)	3757 (62.5)	1941 (32.3)	309 (5.1)		
Other trauma (n = 9455)						
No	7004 (74.1)	5223 (74.6)	1553 (22.2)	228 (3.3)	356.88	<.001
Yes	2451 (25.9)	1332 (54.3)	939 (38.3)	180 (7.3)		

^a^ Participants with a total of 5 or fewer missing values from each of the scales were included in analyses. Therefore, total number of participants varies in the different analyses.

### Factors Associated With Level of Upset

In the first model, we found that higher mean levels of upset were associated with female sex (point estimate, 0.26; 95% CI, 0.20-0.33; *P* < .001), age (point estimate, −0.02; 95% CI, −0.20 to −0.01; *P* < .001), and being exposed to higher numbers of different traumatizing events (point estimate, 0.23; 95% CI, 0.22-0.24; *P* < .001) (Table 2). Using the second model analyzing type of trauma exposure, we found that youths who had been exposed to sexual abuse reported significantly higher levels of upset compared with all other trauma types (point estimate, 0.87; 95% CI, 0.77-0.95; *P* < .001), and 854 participants (58.6%) who had been exposed to sexual abuse reported moderate or high levels of upset. Further, youths who had been exposed to noninterpersonal trauma reported significantly lower levels of upset (point estimate, 0.24; 95% CI, 0.17-0.30; *P* < .001) compared with all other trauma types, and 2250 participants (37.4%) reported moderate or high levels of upset. There was no statistically significant difference in level of upset among youths who had been exposed to domestic violence vs those who had been exposed to community violence (β = 0.01; 95% CI, −0.01 to 0.09; *P* = .80) (Table 3 and Table 4). Using the last model, which included posttraumatic stress symptoms as variables, we found that level of posttraumatic stress symptoms was associated with higher levels of upset (point estimate, 0.05; 95% CI, 0.04-0.50; *P* < .001), and sex and age were no longer statistically significant variables (Table 5).

**Table 2.** Level of Upset by Total Number of Traumas

Variable	Point Estimate (SE) [95% CI]^a^	*P* Value
Age	−0.02 (0.01) [−0.03 to −0.01]	<.001
Female sex	0.26 (0.03) [0.20 to 0.33]	<.001
No. of traumas	0.23 (0.01) [0.22 to 0.24]	<.001

^a^ Analysis was based on 10 008 participants from 40 clinics. The random-effects estimate was 0.17 (95% CI, 0.12-0.24) between clinics and 1.46 (95% CI, 1.44-1.48) within clinics.

**Table 3.** Level of Upset by Type of Trauma

Variable	Point Estimate (SE) [95% CI]^a^	*P* Value
Age	−0.02 (0.01) [−0.03 to −0.01]	<.001
Female sex	0.18 (0.03) [0.12 to 0.25]	<.001
Domestic violence	0.44 (0.04) [0.37 to 0.51]	<.001
Community violence	0.43 (0.03) [0.36 to 0.49]	<.001
Sexual violence	0.87 (0.05) [0.77 to 0.95]	<.001
Noninterpersonal trauma	0.24 (0.03) [0.17 to 0.30]	<.001

^a^ Analysis was based on 8986 participants from 40 clinics. The random-effects estimate was 0.20 (95% CI, 0.15-0.27) between clinics and 1.46 (95% CI, 1.44-1.48) within clinics.

**Table 4.** Significance Testing of Differences in Level of Upset by Trauma Type

Comparison Groups	Difference in Point Estimate (95% CI)	*P* Value^a^
Sexual abuse vs		
Domestic violence	0.43 (0.30 to 0.55)	<.001
Community violence	0.44 (0.32 to 0.56)	<.001
Noninterpersonal trauma	0.63 (0.52 to 0.74)	<.001
Domestic violence vs		
Community violence	0.01 (−0.01 to 0.09)	.80
Noninterpersonal trauma	0.20 (0.10 to 0.30)	<.001
Community violence vs noninterpersonal trauma	0.19 (0.09 to 0.29)	<.001

^a^ Adjusted with Holm correction.

**Table 5.** Level of Upset by Symptoms of Posttraumatic Stress

Variable	Point Estimate (SE) [95% CI]^a^	*P* Value
Age	−0.02 (0.01) [−0.04 to 0]	.05
Female sex	0.04 (0.06) [−0.07 to 0.16]	.44
Posttraumatic stress disorder symptoms	0.05 (0) [0.04 to 0.05]	<.001

^a^ Analysis was based on 3715 participants from 40 clinics. The random-effects estimate was 0.22 (95% CI, 0.15-0.32) between clinics and 1.58 (95% CI, 1.54-1.61) within clinics.

## Discussion

In this study of 10 157 youths who were routinely screened for trauma at youth mental health clinics, we found that 4 of 5 youths reported exposure to at least 1 potentially traumatizing event. Most participants did not find the trauma screening upsetting, with 68.4% reporting no or low levels of upset. To our knowledge, this is the first study that has examined responses to routine trauma screening in a clinical sample of youths. In previous population studies in 2011^21^ and 2014,^20^ 4.6% to 5.7% of participants reported that they became upset by being surveyed. Among participants in the study by Finkelhor et al,^20^ 0.8% said they were very upset, whereas 0.2% of the adolescents in the study by Zajac et al^21^ remained upset after the interview. In our study, 31.2% of participants who had been exposed to trauma and 12.0% of participants who had not been exposed to trauma reported moderate levels of upset, and 5.2% of the participants who had been exposed to trauma and 1.5% of the participants who had not been exposed to trauma reported high levels of upset. This suggests that the level of upset was higher in our population than in the population studies by Zajac et al^21^ and Finkelhor et al.^20^ Although it is not possible to make direct comparisons with the population studies since there may be cultural differences and the study designs were different, the comparison is still interesting. Knowledge about trauma exposure and the participant’s background and ongoing situation in life are important for treatment planning. It is encouraging that the clinical group of youths reported low levels of upset overall. This may be associated with the setting where the youths were asked to complete the questionnaire. They were in the presence of a therapist and in a child-friendly environment where they had been referred to receive help. They may have expected to be asked difficult questions and thus were prepared to answer questions about their lives. In a population-based study, the context may be quite different. The fact that most of the youths who had not been exposed to trauma did not report being upset is an important clinical finding because clinicians may be reluctant to administer unnecessary questions to youths, particularly if the questions could be upsetting. For youths who have been exposed to trauma, clinicians may be more willing to accept some level of upset because disclosure of trauma is essential to assess possible PTSD symptoms and to customize a treatment plan accordingly. In addition, clinicians are trained to manage emotional distress, and feedback from the screening can be used to validate experiences and provide mental health education.

A total of 79.1% of participants reported exposure to at least 1 potentially traumatizing event. For example, 26.8% reported exposure to domestic violence and 14.4% reported exposure to sexual abuse. These numbers seem generalizable, given the population prevalence of violence and sexual abuse in Norway that suggest that 4.6% of girls experience sexual abuse before the age of 18 years and 6.9% of youths (10.2% girls and 3.5% boys) have had sexual contact with another person at least 5 years older than the youth and in which the sexual contact included an attempt of penetration.^25^ Furthermore, 5% of youths report exposure to physical abuse in their family, and 10% report witnessing domestic violence.^25^

Additionally, we found that there were differences in levels of upset based on trauma type. Youths who had been exposed to interpersonal trauma reported higher levels of upset than those who had been exposed to noninterpersonal trauma. For example, among participants who had been exposed to noninterpersonal violence, 37.4% reported moderate or high levels of upset. In comparison, 58.6% of participants who had been exposed to sexual abuse reported moderate or high levels of upset. This may be because interpersonal trauma often leads to more difficulties regulating emotions and also to higher levels of interpersonal problems and distrust.^26^ Youths who had been exposed to sexual abuse reported significantly higher upset scores than youths exposed to all other trauma types. A 2016 study^19^ found that exposure to most types of serious violence, including sexual abuse, was associated with more shame than exposure to other types of traumas. It may be that shame adds to the emotional upset associated with being reminded of abuse. It may also be that these traumas are the ones most likely to not have been disclosed before and that the screening is their first disclosure. For patients who have not previously disclosed trauma, not knowing what to expect from disclosure may lead to apprehension and heightened alert.

Higher levels of PTSD symptoms were associated with higher levels of upset. This is also to be expected, as hypervigilance, re-experiencing, and strong negative emotions when reminded of the trauma are part of the PTSD symptom criteria.^16^ The trauma screening may act as a reminder and elicit posttrauma reactions and fear. These youths are likely to experience trauma reminders on a daily basis, causing heightened upset. A child with trauma, like any child, needs someone who listens to their experiences and who validates their emotions and reactions. When trauma experiences are identified in a clinical context, these youths may receive treatment to help alleviate this upset. However, screening for trauma exposure is only the first step in treatment planning. Not everyone who is exposed to trauma develops posttrauma reactions that need to be treated. After the identification of trauma experiences, posttrauma responses and other comorbid conditions need to be evaluated with a thorough assessment using multiple informants. Most importantly, trauma screening should be followed by a validating conversation between the patient and the clinician in which possible actions in case of ongoing and threatening traumatization are discussed as well.

### Limitations

A limitation in this study is that we did not ask what kind of screening questions were most upsetting or why participants felt upset. For example, in a study by Kassam-Adams and Newman^27^ involving reactions of children participating in a study of acute posttraumatic stress after a recent injury, they found that few children reported regret, sadness, or upset from participation and nearly 9% felt bored by the questions. It may be that some participants in our study were upset for other reasons, such as being upset for having to do the screening. Further studies could examine reasons for being upset, as this would be helpful to guide clinicians. Also, we did not ask about positive reactions related to screening. It may be that some participants who reported becoming upset also were relieved or content that others were interested in their life experiences and cared enough to ask. Although the screening recommendation was that therapists do the screening as an interview, we do not have information on whether participants completed the screening inventory themselves or whether the therapist asked the questions in person. Future research should collect this information systematically to find out what ways of screening are least distressing and most valid.

## Conclusions

Identifying trauma exposure is a prerequisite to diagnosing PTSD and treatment planning. To our knowledge, this is the first study to examine self-reported upset associated with trauma screening among a real-world clinical population of youths. Taken together, the results show that although most youths in this clinical sample did not report high levels of upset after trauma screening, many reported moderate levels of upset. Those who reported interpersonal trauma in general and sexual abuse in particular reported the highest upset scores. There is general agreement that youths with PTSD symptoms benefit more from treatments addressing their traumatic experiences compared with more general treatments.^13,28^ Routine trauma screening is necessary to be able to offer appropriate services, as many traumas, especially interpersonal traumas, might be associated with high levels of secrecy. The negative cumulative effect of trauma is well documented^29,30^; hence, early identification of trauma exposure may also have preventive effects. The results from this study could be used to support therapists and health systems to routinely screen for trauma when effective treatments are available once trauma is disclosed. This could help bridge the gap between research-supported international guidelines on routine trauma screening and the real-world practice of youth trauma treatment.
